# Special strategies and management of urological diseases during the COVID-19 Pandemic: initial experiences from a Medical Center of China

**DOI:** 10.1590/S1677-5538.IBJU.2020.S102

**Published:** 2020-07-27

**Authors:** Wei Chen, Xiao-Meng Wang, Guang-Qing Fu, Xiang Zeng, Cui-Ping Wu, Yong Liang, Jian-Hui Liu, Jeremy Yuen-Chun Teoh

**Affiliations:** 1 Zigong Fourth People's Hospital Department of Urology Sichuan China Department of Urology, Zigong Fourth People's Hospital, Sichuan, China; 2 Zigong Fourth People's Hospital Department of Science and Education Sichuan China Department of Science and Education, Zigong Fourth People's Hospital, Sichuan, China; 3 Zigong Fourth People's Hospital Department of Hospital Infection Sichuan China Department of Hospital Infection, Zigong Fourth People's Hospital, Sichuan, China; 4 The Chinese University of Hong Kong SH Ho Urology Centre Department of Surgery Hong Kong China Department of Surgery, SH Ho Urology Centre, The Chinese University of Hong Kong, Shatin, Hong Kong, China

**Keywords:** COVID-19 [Supplementary Concept], Pandemics, Urology

## Abstract

Although urological diseases are not directly related to coronavirus disease 2019 (COVID-19), urologists need to make comprehensive plans for this disease. Urological conditions such as benign prostatic hyperplasia and tumors are very common in elderly patients. This group of patients is often accompanied by underlying comorbidities or immune dysfunction. They are at higher risk of COVID-19 infection and they tend to have severe manifestations. Although fever can occur along with urological infections, it is actually one of the commonest symptoms of COVID-19; urologists must always maintain a high index of suspicion in their clinical practices. As a urological surgeon, how we can protect medical staff during surgery is a major concern. Our hospital had early adoption of a series of strict protective and control measures, and was able to avoid cross-infection and outbreak of COVID-19. This paper discusses the effective measures that can be useful when dealing with urological patients with COVID-19.

## INTRODUCTION

Coronavirus disease 2019 (COVID-19) has become a global pandemic since the beginning of 2020. The WHO announced COVID-19 as a public health emergency (PHEIC) of international concern on January 30, 2020 (1). COVID-19 has caused many close contacts to be infected because early symptoms can be subtle; patients mainly have dry cough and fatigue, and some even present without any symptoms; this imposes substantial challenges for prevention of viral transmission (2, 3). For urology departments, both outpatient clinics and wards are high-risk areas where the virus can be exposed and spread. According to the current global case statistics, a large number of medical staff has been infected, and some resulted in mortality. Most of the deaths caused by COVID-19 are in middle-aged and elderly patients with chronic diseases (such as tumors, cirrhosis, hypertension, coronary atherosclerotic heart disease, diabetes, and Parkinson's disease) (4). Urological conditions, such as benign prostate hyperplasia and tumors, are very common in elderly patients, who unfortunately are high-risk individuals due to underlying comorbidities and immune dysfunction (5, 6). During this critical time period of COVID-19 epidemic, we must strengthen the protection of doctors, nurses and patients in urology departments (7).

China has proactively taken strict measures to address this global pandemic at a very early stage. COVID-19 has been included as a class B infectious disease under the Law of China on the Prevention and Control of Infectious Diseases and as a class A infectious disease in terms of the planned management strategies. In the process of anti-epidemic strategy implementation, the National Health Commission of P.R. China has continuously issued and updated seven versions of the guidelines for the prevention and control of COVID-19. As of 24:00hrs on April 27, a total of 82,836 cases of COVID-19 were diagnosed in China, of which 4,633 cases died. Currently, there are 50 severe cases out of 648 confirmed cases (8). Although China's epidemic situation has not yet completely resolved, it has achieved staged victory in the control of this epidemic with substantial efforts by solid unity throughout the nation.

In this paper, we introduce the initial experiences of the special management strategies of urological diseases during the COVID-19 pandemic in China.

### The prevention and control strategies of our hospital

#### Rearrangement of the hospital area

To prevent the potential infection risk resulting from unscreened people entering the hospital, we have set pre-examination and triage locations in front of the hospital. Qualified clinicians were arranged in rota to participate in the 24-hour work at this position to monitor the temperature of each person entering the hospital and to inquire for any upper respiratory symptoms and epidemiological history. People with a history of fever or contact with the epidemic area were mandated to receive consultation at the fever clinic.

The hospital had a strict division of personnel. There were six access channels for staff, common patients, and fever patients started at the beginning of January. Additional fever clinics and fever wards were temporarily established to cope with the sudden increase in fever patients. We also set up special equipment and channels for fever patients to receive computed tomography, ultra-sound, and other auxiliary examinations.

#### Diagnosis and treatment items and department administration

According to the needs of epidemic prevention and control, the hospital suspended outpatient services and provided emergency dental, ophthalmological, and otolaryngological services to minimize the risk of cross-infection while the number of confirmed cases in China continued to increase. In terms of urological investigations, we suspended items such as urodynamic testing, cystoscopy, andrology examinations, and extra-corporeal shock wave lithotripsy, based on the urgency of urological conditions.

#### Management of the urology clinic

**Figure 1:**
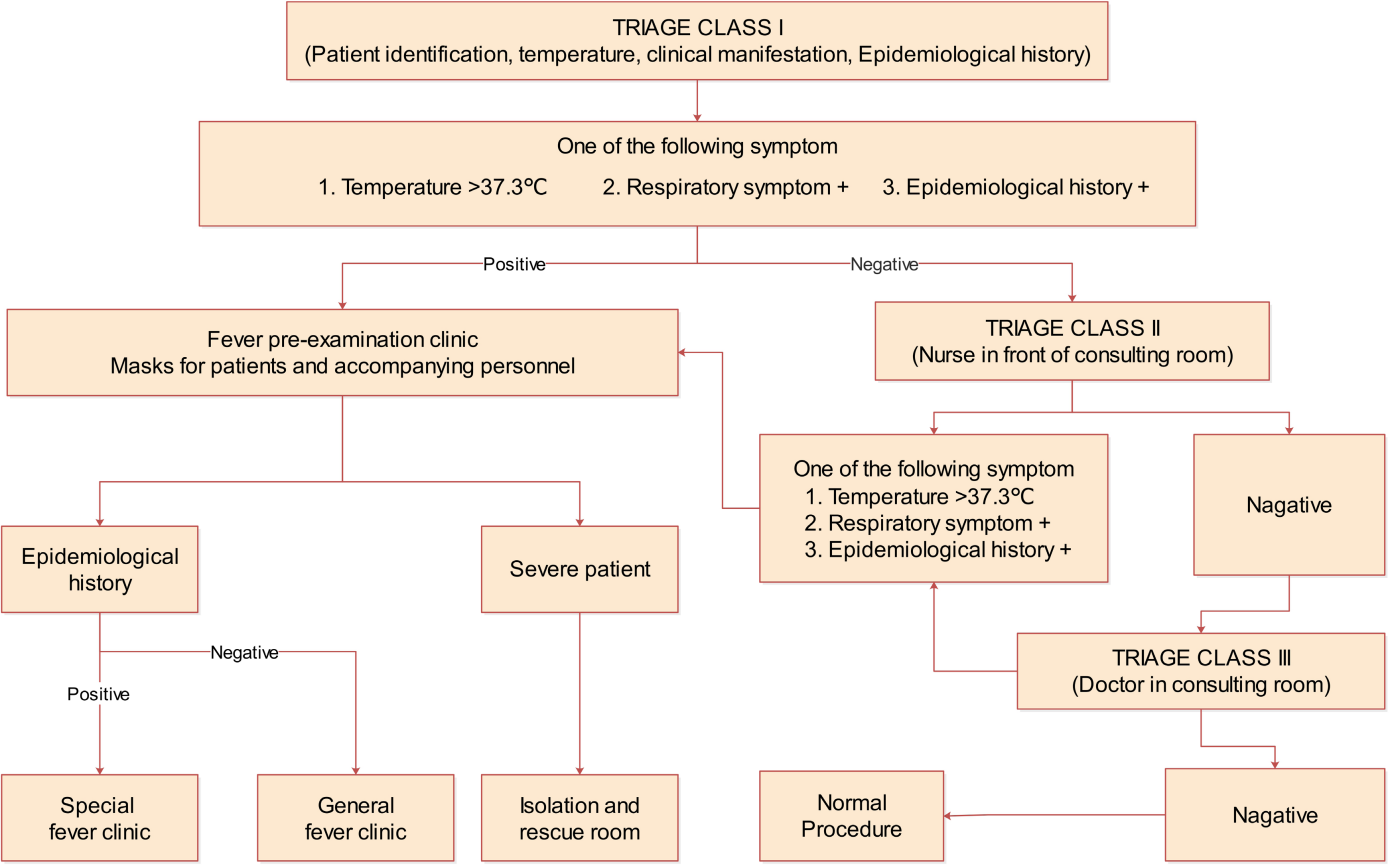
Three levels of pre-examination for urology clinic.

A three-level arrangement of pre-examination and triage was adopted for the management of the urology clinic (Figure-1). Everyone entering the outpatient building was required to wear a mask and undergo a temperature measurement. Those with body temperatures greater than 37.3°C were not allowed to enter the clinic.

#### 1 - For medical staff

A triage nurse was required to first screen the patient for an epidemiological history and to fill out a registration form. The clinic doctor asked for the patient's medical history in detail and recorded the outpatient medical record faithfully. All medical staff needed to strengthen their awareness of hand hygiene and to strictly follow the requirements from the hospital. To avoid cross--infection, staff could not touch their faces if they did not wash or disinfect their hands. If the patient was suspected to have COVID-19, the “Suspicious and Confirmed Case Report Requirements and Procedures” guidelines proposed by the hospital were followed.

#### For patients

In addition to the examinations that required close contact, patients needed to maintain a certain distance from their accompanying family member and from the medical staff. Strict implementation of “one person, one clinic, one room” was required to avoid overcrowding in the waiting area.

#### Management of urology wards

**Figure 2:**
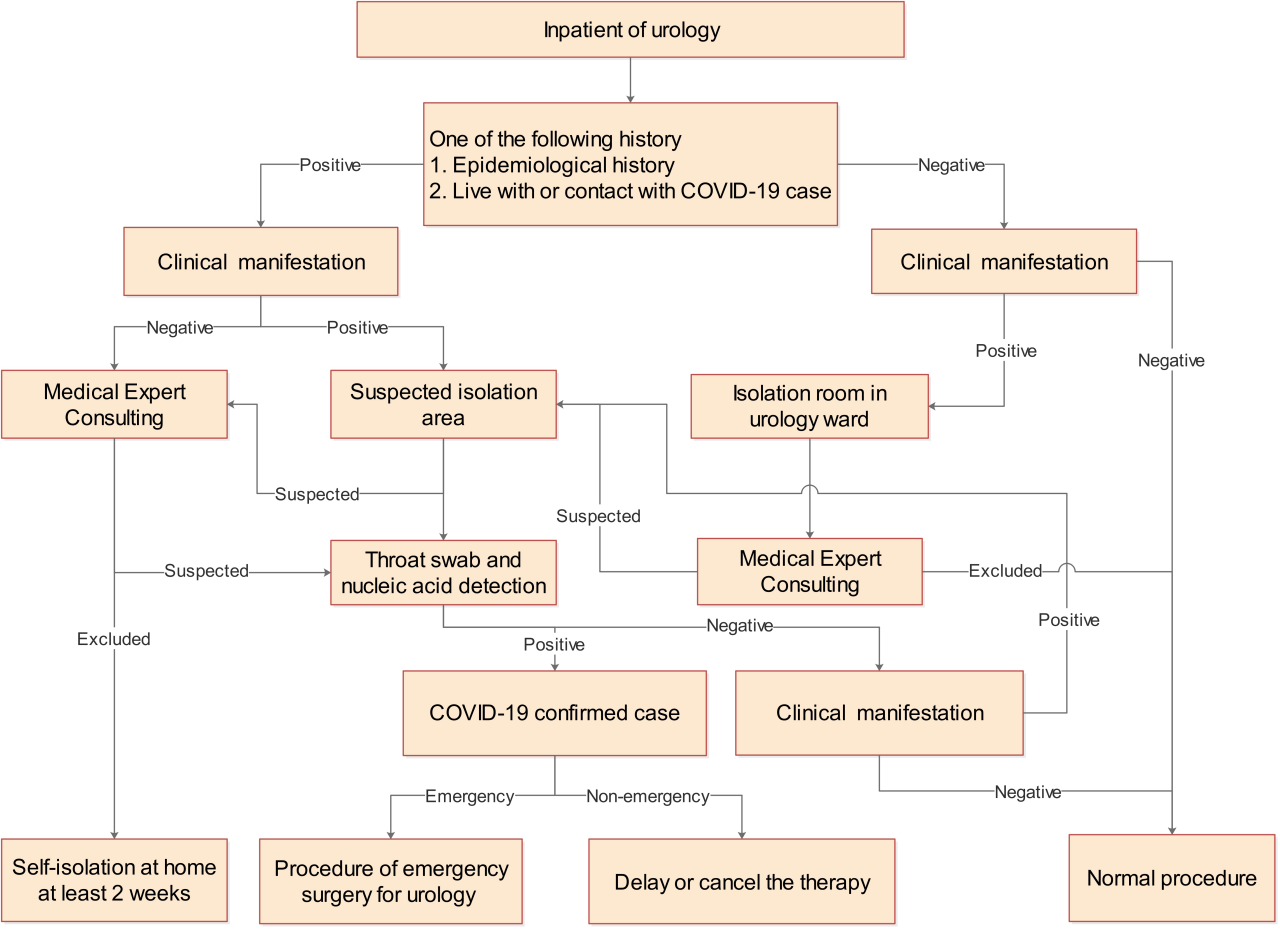
General screening procedures for patients in urology ward.

There were many people in the urology ward. We adjusted the ward setting, restricted the entry and exit of patients and medical staff, and made use of the Internet technology to carry out all ward management. The specific measures were as follows:

1. **Online diagnosis and treatment:** we reduced the admission of inpatients if they were non-emergencies. Urologists could participate in online consultations to answer questions for patients, which significantly relieved the pressure of the front-line medical staff. To reduce the chance of cross-infection caused by patients coming to the hospital for a consultation, urologists could also strengthen their follow-up management of discharged patients through self-developed mobile apps.
2. **Control of visitors:** we restricted the number of outside visitors allowed to enter the ward. Generally, escort or visiting outside a certain time was not allowed. No more than 1 person was allowed, and no more than 15 minutes of visiting time per day per patient was allowed.
3. **Noncontact delivery:** meal delivery or courier delivery from outsiders were not allowed to enter the ward. Ward control points and take--out delivery corners were set up at the entrance of the inpatient building.
4. **Ward management:** all the air conditioners in the ward were turned off. Double access of passages and elevators without crossing the ward was formulated for medical staff and patients. There were 1-2 isolation cubicles in each ward. Once there was a suspected patient who needed to be quarantined on the spot, the patients in the same cubicle were arranged for isolation to receive expert consultation and they will enter a screening process (Figure-2).
5. **The implementation of protective measures was reinforced by nurses on a daily basis:** Behaviors such as poor hand hygiene, irregular use of masks, people gathering and lack of cough etiquette were corrected, followed by a thorough explanation and correct demonstration. The responsible team leader conducted two special inspections every morning and afternoon for patients and visitors/accompanying personnel.
6. **All urologists** were informed to stop taking vacations as well as leaving the city. Those who had not been arranged for work should rest at home. Urology staff received training to strengthen risk awareness cultivation under the requirements of the hospital. The staff was also required to participate in the relevant training exercises and pass the assessment organized by the hospital.

### Treatment suggestions for urological diseases

#### Management of patients with fever in urology

Fever and pneumonia are common in urology patients and the differential diagnosis of COVID-19 should be considered. Patients with fever and urological symptoms, and without any respiratory symptoms, should be differentially diagnosed with urological infections. Patients with fever and respiratory symptoms should be screened according to the COVID-19 screening procedure. Moreover, necessary isolation and disinfection should be performed. In addition, any suspected cases should also be reported to the hospital infection management department and consulted by an expert group to provide assistance in diagnosis and treatment.

In addition, the majority of urology patients are of advanced age. CT examination of the lungs may identify asymptomatic pneumonia or febrile pneumonia. COVID-19 infection needs to be screened in these patients, and epidemiological history must be enquired to avoid the risk of asymptomatic infection. For those who are asymptomatic at the hospital but then developed symptoms during the hospitalization period, COVID-19 nucleic acid test is required and should be repeated if necessary after reporting to the hospital.

#### Management of patients receiving surgery

First, to avoid cross-infection, we should try to limit elective surgery as much as possible during the epidemic. Emergency or urgent surgeries should be considered under special protective circumstances. Confirmed cases should be transferred to designated hospitals for further processing. Second, when urological procedure has to be performed on suspected cases or confirmed cases, medical staff should work with protective clothing, shoes, and head coverings and should disinfect the items used by the patients, marking the designated place and time.

**Figure 3:**
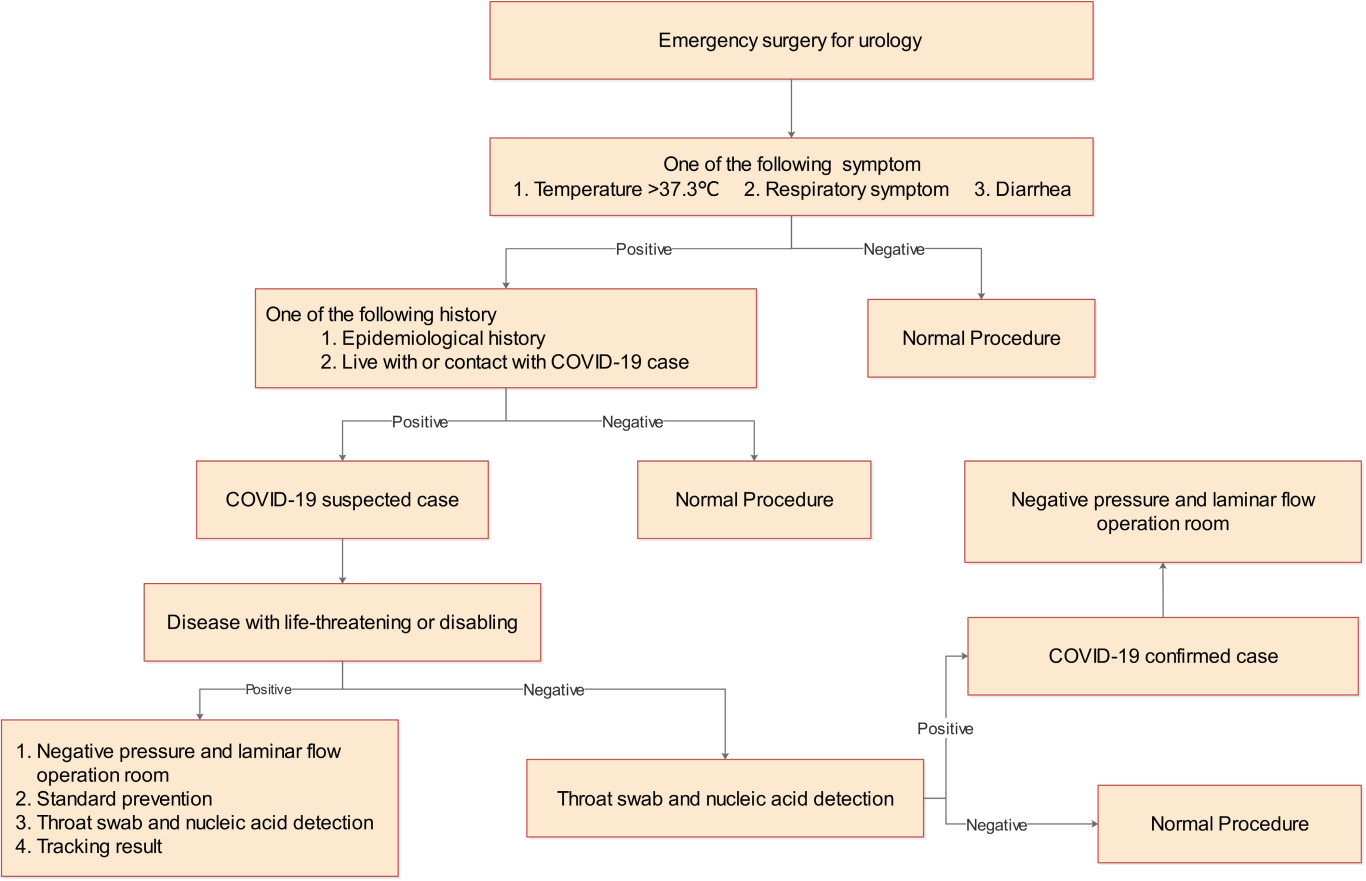
Procedure for emergency surgery of urology during COVID-19 pandemic.

For patients who have life-threatening urological emergencies requiring emergency surgeries, and when COVID-19 testing results are not available, the operation needs to be performed in a negative-pressure theatre or infection operating room after consultation with an expert group. The procedure for the arrangements of emergency surgery is shown in Figure-3. The patient should enter the operating room with a mask. The medical staff should adopt level three protection with surgical gowns and shoe covers, protective clothing, goggles or face screens, and N95 masks (European mask standard EN149: 2001 (FFP2). At the same time, minimal use of items and restriction of medical personnel should be considered. After the operation, the protective equipment must be removed in order. The in-time cleaning and disinfection of the operating room is necessary. If the patient is diagnosed with COVID-19 after surgery, a detailed report to the medical administration department and self-monitoring with isolation for 2 weeks for the participating surgical staff are required.

#### Collection of stool specimens

Stool specimens are commonly collected for various laboratory investigations. Due to the existing evidence that COVID-19 viral RNA can be detected in stool, the stool samples should be carefully handled during the collection and submission period (9, 10). Viral RNA has also been detected in urine samples, but the rate is very low. Any leakage or cross-infection can be avoided by stringent procedures.

The preparation before stool collection for patients with confirmed or suspected COVID-19 should include a closed sterile container with stool, a closed bag for all patients and a disposable urine cup if probable for female patients. The collected stool should be placed in a sterile, closed container. The samples must be marked for patients with COVID-19 and must indicate the patient's information that needs to be collected. Leaving the stool outside the container is forbidden during the collection process. If this really happens, immediate wiping with a sterile towel and ethanol more than 5 times before putting the sample in a closed bag are required. The samples should be stored at 4 degrees centigrade if long-distance transshipment is needed. The sample should be kept at 4 degrees centigrade if the detection time is within 5 days; otherwise, 70 degrees below centigrade is required. To reduce the possibility of exposure to pathogens, all samples collected for laboratory testing should be considered potentially infectious. Health care workers collecting and transporting clinical samples should strictly abide the guidelines for infection prevention and control. In addition, proper communication should be carried out to ensure proper specimen collection and to guarantee the lowest possible risk of infection to the collector.

## CONCLUSIONS

“Prevention is better than cure” – it is the golden rule for any infectious disease at any time. The patients seen by urologists are mostly elderly people, who are the frequent population suffering from severe diseases. We must strengthen protection and health education in order to fight against this disease.
